# Increased frequency of intentional weight loss associated with reduced mortality: a prospective cohort analysis

**DOI:** 10.1186/s12916-020-01716-5

**Published:** 2020-09-17

**Authors:** Erik A. Willis, Wen-Yi Huang, Pedro F. Saint-Maurice, Michael F. Leitzmann, Elizabeth A. Salerno, Charles E. Matthews, Sonja I. Berndt

**Affiliations:** 1 Division of Cancer Epidemiology and Genetics, National Cancer Institute, National Institutes of Health, Department Health and Human Services, Bethesda, MD USA; 2 Center for Health Promotion Disease Prevention, University of North Carolina at Chapel Hill, Chapel Hill, NC USA; 3 Department of Epidemiology and Preventive Medicine, University of Regensburg, Regensburg, Germany

**Keywords:** Obesity, Weight loss, Mortality, Prospective cohort

## Abstract

**Background:**

Due to the high prevalence of obesity and the difficulty in maintaining weight loss, repeated bouts of weight loss are a common occurrence. However, there are inconsistencies in epidemiological studies regarding repetitive weight fluctuations being associated with increased risk of mortality. Therefore, the purpose of this prospective cohort analysis was to determine the long-term association of the frequency of weight loss attempts on mortality.

**Methods:**

This prospective cohort study used data collected from adult AARP members living in 6 states (California, Florida, Louisiana, New Jersey, North Carolina, or Pennsylvania) or 2 metropolitan areas (Atlanta, Georgia, or Detroit, Michigan) and participating in the National Institutes of Health–AARP Diet and Health Study between 2004 and 2006. Self-reported data were analyzed for 161,738 middle-aged adults. During an average 7 years of follow-up, 21,194 deaths were recorded. Hazard ratios of all-cause, cardiovascular, and cancer mortality were estimated adjusting for demographic, lifestyle, and behavioral risk factors.

**Results:**

Increased frequency of weight loss attempts of at least five pounds was associated with lower mortality (*p*_trend_ < 0.010). Multivariate hazard ratios (95% confidence intervals) for all-cause death among individuals who successfully attempted weight loss compared with those who did not make any attempts were 0.94 (0.90–0.98) for 1–2 attempts, 0.96 (0.91–1.01) for 3–4 attempts, 0.91 (0.85–0.96) for 5–6 attempts, 0.91 (0.85–0.98) for 7–8 attempts, 0.87 (0.80–0.95) for 9–10 attempts, and 0.88 (0.82–0.94) for 11+ attempts. Similar results were noted for men and women, participants with healthy weight and overweight/obesity, and even among those who gained weight over time. Protective associations were also observed for deaths due to cardiovascular disease and cancer.

**Conclusions:**

Increased frequency of intentionally losing at least five pounds in mid-life was associated with a lower risk of future death. Repeated attempts with moderate amounts of weight loss may provide benefit in terms of longevity.

**Trial registration number:**

ClinicalTrials.gov number, NCT00340015

## Introduction

With the rise in obesity prevalence worldwide and its negative impact on health [1–4], weight management has become a key public health focus. Studies have consistently shown health benefits associated with clinically significant weight loss (3–5%) [5–8], and it is recommended that individuals who are classified as overweight/obese lose weight gradually (~ 1–2 lbs/week) [9] using evidence-based approaches [10]. The proportion of US adults who report intentionally losing weight is substantial and has increased from 43% in 2007 to 49% in 2016 [11]. However, approximately 65–80% of individuals who intentionally achieve clinically meaningful weight loss will regain some, if not all, of the weight lost within 12 months after stopping treatment [12, 13]. As a consequence, repeated bouts of intentional weight loss is a common occurrence [14], with some reporting as many as 50 weight loss attempts over their lifetime [15],

Early epidemiological studies suggested that repetitive weight fluctuations are associated with increased risk of mortality [16–20], several chronic diseases [21], and poor mental health [22], which called into question the benefits of recommending adults with overweight/obesity to lose weight [23]. However, recent evidence suggests there is no association between repeated bouts of weight loss and negative health outcomes, body composition, or future obesity [24, 25]. Such inconsistencies may be due to lack of information on total volume of weight lost and/or type of weight loss (e.g., intentional versus unintentional) or failure to control for important confounders (e.g., smoking, body mass index [BMI], overall weight trajectories). It is particularly important to differentiate unintentional from intentional weight loss, since weight loss due to illness may be reflective of poor health [26], which could confound the association. To clarify these issues, we evaluated the association of frequency of intentionally losing at least five pounds with all-cause and cause-specific mortality in middle-aged adults in the National Institutes of Health (NIH)–AARP Diet and Health Study (ClinicalTrials.gov number, NCT00340015).

## Methods

### Study population

As described previously [27], the NIH-AARP Study is a prospective cohort of 566,407 men and women aged 50–71 years who returned a baseline questionnaire (BQ) eliciting information on demographic and health-related behaviors between 1995 and 1996. A second questionnaire (Risk Factor Questionnaire; RFQ), which collected more detailed information about anthropometric characteristics at different ages, was sent subsequent to the BQ in late 1996 to respondents still living in the study area and not having prevalent cancer of the prostate, breast, or colon. A third questionnaire (Follow-up Questionnaire; FQ) asking detailed questions about lifestyle behaviors, including intentional weight loss, was completed by 313,363 participants in 2004–2006. The NIH–AARP Study was approved by the Special Studies Institutional Review Board of the National Cancer Institute, and all participants gave written informed consent by completing and returning the questionnaire.

Of the 419,154 non-proxy respondents who returned either the RFQ or FQ, we excluded those without FQ data (*n* = 124,284). While exact reasons for not completing FQ are unknown, from death record ascertainment, we are able to determine only 21% of those excluded died prior to FQ and 81% had self-reported their health condition at time of RFQ as Good-Excellent. Furthermore, we excluded those with extreme frequency and volume of weight loss (twice the interquartile range; *n* = 1315) and those that were underweight or with extreme BMI values (< 18.5 kg/m^2^ or > 60.0 kg/m^2^ on the RFQ or FQ) or did not have BMI for at least two time points (*n* = 24,799). Finally, we excluded those with missing information on intentional weight loss (*n* = 107,018). The analytic cohort included 161,738 participants (100,416 men and 61,322 women). The socio-demographics and behavioral characteristics of this analytic cohort were broadly similar to all potentially eligible participants (data not shown).

### Assessment of weight loss frequency

The frequency of losing at least five pounds in the past 20 years was assessed by self-report on the FQ (Additional file 1: Fig. S1), by summing the total number of times participants reported losing weight using the mid-point value in each frequency category. Participants were then categorized by frequency of weight loss (never, 1–2, 3–4, 5–6, 7–8, 9–10, and 11+ attempts). The average volume (i.e., lbs) of weight loss per attempt was calculated by dividing total volume of weight loss (based on summing the mid-point values for loss) over all attempts by the total frequency of attempts.

### Assessment of historical weight, BMI, and other covariates

Participants reported their height and current weight on both the BQ and FQ, and historical weights at age 35 and 50 on the RFQ, from which we calculated age-specific BMIs (kg/m^2^). To determine the participant’s starting BMI that preceded the 20-year intentional weight loss period that ended at FQ, we used the “historical” BMI at either age 35 or 50, whichever was closest to the participant’s age at the FQ minus 20 years. Historical BMI was categorized as healthy weight (18.5–< 25.0 kg/m^2^), overweight (25.0–< 30.0 kg/m^2^), obese (30.0–< 35.0 kg/m^2^), and severely obese (≥ 35.0 kg/m^2^). Other covariates, such as physical activity and sedentary time, were based on self-reported data collected on the FQ.

### Death ascertainment

Mortality was compiled via the National Death Index, through December 2011. Causes of death were classified using the International Classification of Diseases codes. End points for our analysis were all-cause-, cancer- (ICD: 140-208, 238.6, C00-C97), and cardiovascular-mortality (ICD: 390-398, 401-404, 410-429, 440-448, I00-I13 I20-I51, I60-I78). Vital status ascertainment in this cohort was > 95% [28].

### Statistical analysis

Individual participant linear slopes for BMI were derived using linear mixed models with fixed and random effect for time. Models were run by sex to account for sex-specific differences in weight across time. Participants were then classified as weight losers (slope < 25th percentile), weight maintainers (slope 25th to 75th percentile), and weight gainers (slope ≥ 75th percentile). Additionally, due to weight change of as little as 3% being associated with changes in health outcomes [5–8], sensitivity analysis of participants categorized as weight gainers (>+ 3% BMI change), maintainers (− 3% to + 3%), or losers (<− 3%) were completed. Results of analyses that included weight change as ± 3% did not differ meaningfully; therefore, models with weight change categorized by linear slopes are reported (see Additional file 1: Table S6).

Cox proportional hazard models, with age as the underlying time metric, were fit to estimate hazard ratios (HRs) and 95% confidence intervals (CIs) for mortality across increasing frequency of successful weight loss attempts. Participants were followed prospectively from the date of the FQ completion (i.e., on which individuals reported previous weight loss attempts) to either death or end of follow-up (December 31, 2011), whichever came first.

We assessed mortality risk in two models: (1) adjusting for age, sex, race/ethnicity, education level, healthy eating index, physical activity, sedentary time, smoking, number of chronic diseases, overall health, marital status, age at retirement, age at menopause (for women) and historical BMI and (2) additionally adjusting for weight change categories (i.e., gainer, maintainer, loser). Tests of linear trends were conducted by modeling the median value from each frequency of weight loss attempt category as a continuous variable, after exclusion of the unintentional weight loss group. In secondary analyses, we stratified by median age at start of intentional weight loss period (< 51, ≥ 51 years), sex, historical BMI (≥ 18.5–< 25.0 kg/m^2^, ≥ 25.0 kg/m^2^), weight change categories, and smoking status (never, former, current). Missing data for covariates were treated as a separate category and included in the models. Because competing risks may play a role in estimating cause-specific mortality, sub-distribution hazard models were evaluated for cancer and CVD specific mortality [29].

As a sensitivity analysis, to evaluate the possible impact of excluding participants with missing data on intentional weight loss, we used the multiple imputation method in which we regressed these measures on a number of other individual-level variables, including age, sex, race/ethnicity, education level, healthy eating index, physical activity, sedentary time, smoking, chronic diseases, overall health, marital status, age at retirement, age at menopause (for women), historical BMI, and weight change [30]. Imputed estimates and variance from 10 imputed datasets were combined to obtain the final estimated HRs and 95% confidence intervals (CIs).

To evaluate associations with total weight lost volume over 20 years and frequency of successful weight loss attempts, we jointly classified frequency and total volume of weight loss categories (5–50 lbs, 50–100 lbs, 100–150 lb., and 150+ lbs). All analyses were conducted using SAS version 9.4 (SAS Institute Inc., Cary, NC).

## Results

In the analytic cohort (*N* = 161,738), mean age at the start of the weight loss observation period was 51 years (range, 39–63 years). Approximately 62% of participants were male, 93% non-Hispanic white, and 6% current smokers. Of those reporting intentional weight loss, the median number of attempts of at least five pounds over the past 20 years was 4 (range 2–23) with an average of ~ 11 pounds lost per attempt. Participants with more frequent attempts were more likely to be female, former/current smokers, overweight/obese at the beginning of the weight loss period, have 5+ medical conditions and self-report their health as fair/poor compared those with no or few attempts (Table 1); however, there were no substantial differences in levels of physical activity, sedentary behavior, or diet quality (Table 1). Across the median 7.1 years of follow-up, 21,194 deaths occurred.

**Table 1:** Socio-demographic and behavioral characteristics of the participants

		Frequency of weight loss attempts							
	Analysis cohort (*n* = 161,738)	Unintentional WL (*n* = 26,178)	Never (*n* = 44,054)	1–2 (*n* = 33,114)	3–4 (*n* = 19,465)	5–6 (*n* = 13,537)	7–8 (*n* = 9159)	9–10 (*n* = 6054)	11+ (*n* = 10,177)
	%	%	%	%	%	%	%	%	%
**Age at FUQ (yrs.; mean [SD])**	70.7 [5.3]	72.2 [5.0]	71.3 [5.3]	70.7 [5.3]	70.2 [5.3]	69.7 [5.3]	69.5 [5.3]	68.9 [5.2]	68.3 [5.1]
**Average weight loss per attempt (lbs; mean [SD])**	6.1 [6.9]	0	0	9.4 [6.8]	10.9 [5.9]	11.6 [4.9]	11.3 [4.7]	11.4 [3.8]	13.3 [3.1]
**Female (%)**	37.9	42.8	25.1	38.3	40.7	42.5	44.0	48.2	56.5
**Weight change**									
*Weight losers*	28.7	100.0	0.0	28.7	22.7	19.5	17.9	16.1	10.6
*Weight maintainers*	50.7	0.0	79.6	52.8	54.3	52.4	51.2	48.0	40.5
*Weight gainers*	20.6	0.0	20.4	18.6	23.0	28.0	30.9	35.8	48.9
**Race/ethnicity (%)**									
*Non-Hispanic White*	92.6	92.5	91.2	93.2	93.1	93.4	93.4	94.3	93.2
*Non-Hispanic Black*	3.4	2.4	3.9	3.3	3.3	3.5	3.3	3.0	3.7
*Hispanic*	1.6	1.5	2.0	1.4	1.4	1.5	1.4	1.0	1.3
*Asian*, *PI or AI/AN*	1.5	2.7	1.8	1.2	1.2	0.7	1.0	0.9	0.7
*Missing*	1.0	0.9	1.1	0.9	1.0	0.9	0.9	0.9	1.1
**Education level (%)**									
*Less than 8 years*, *8–11 yrs.*	3.9	3.9	5.6	3.4	2.9	2.9	3.2	3.1	3.0
*12 yrs. or completed high school*	18.0	18.2	20.5	17.4	16.6	16.4	15.2	15.9	17.0
*Post-high school*	9.8	9.5	10.8	9.3	9.3	9.2	8.9	9.8	9.7
*Some college*	23.0	21.5	22.1	22.8	23.1	23.7	24.8	26.0	27.3
*College and post graduate*	43.3	44.9	38.5	45.3	46.3	45.8	46.1	43.7	41.1
*Missing*	2.1	2.1	2.4	1.9	1.9	2.0	1.8	1.5	1.8
**Starting BMI (%)**									
*18.5*–*24.9 kg/m*^***2***^	38.3	52.6	42.2	38.4	34.0	29.7	28.9	24.8	20.5
*25.0*–*29.9 kg/m*^***2***^	25.2	13.8	24.5	26.4	29.0	30.7	31.3	31.8	29.0
*30.0*–*34.9 kg/m*^***2***^	4.8	1.3	3.0	4.5	5.4	7.3	7.9	9.7	12.8
*35.0+ kg/m*^***2***^	31.7	32.4	30.3	30.7	31.6	32.3	31.9	33.7	37.7
**Reported medical conditions (%)**									
*None*	6.1	8.6	6.9	5.7	5.3	4.7	4.4	4.1	3.1
*1 to 2*	33.8	39.1	35.8	34.2	31.6	30.1	29.9	28.3	26.1
*3 to 4*	36.7	34.0	36.1	37.8	38.2	37.5	38.5	37.0	37.6
*5+*	23.4	18.3	21.2	22.3	24.9	27.7	27.2	30.6	33.1
*Missing*	0.03	0.02	0.04	0.03	0.01	0.01	0.02	0.02	0.05
**Physical activity (Met-hrs./day [quintiles]; %)**									
*0 to 17.9*	24.9	22.4	26.9	24.0	23.6	24.7	23.6	25.2	28.6
*17.9 to 29.3*	23.3	22.9	22.9	23.7	24.4	23.5	23.3	22.2	22.7
*29.3 to 48.4*	22.7	22.8	21.7	23.9	23.4	23.1	23.3	23.2	20.7
*48.4 to 78.5*	19.4	20.9	18.7	19.2	19.5	19.6	20.1	20.1	18.1
*78.5+*	9.1	10.1	9.0	8.6	8.6	8.8	9.3	9.0	9.6
*Missing*	0.7	0.9	0.8	0.7	0.5	0.4	0.4	0.3	0.5
**Sitting time (hrs./day [quintiles]; %)**									
*0.0 to 4.5*	23.5	29.3	24.5	24.0	22.1	20.1	19.7	18.1	16.2
*4.5 to 6.5*	45.6	44.0	45.2	46.2	47.1	47.5	46.2	45.7	43.1
*6.5 to 8.5*	17.8	14.4	16.8	17.4	18.5	19.6	19.6	21.3	24.3
*8.5 to 12.5*	2.5	2.2	2.5	2.5	2.3	2.6	3.0	3.0	3.4
*12.5+*	2.9	2.2	3.0	2.5	2.7	2.7	3.7	3.6	4.7
*Missing*	7.8	8.0	8.0	7.5	7.4	7.6	7.8	8.3	8.2
**HEI-2015 total score (quintiles; %)**									
*24.0 to 60.0*	21.4	20.5	27.0	19.9	18.5	18.1	18.3	18.0	19.4
*60.0 to 66.3*	20.4	18.6	21.8	19.7	20.4	20.1	20.3	20.3	21.1
*66.3 to 71.2*	19.8	18.7	19.0	20.1	20.6	20.7	20.7	20.9	20.9
*71.2 to 76.2*	19.4	19.3	16.9	20.0	20.7	21.4	20.8	21.2	20.9
*76.2+*	19.0	22.9	15.4	20.2	19.7	19.8	19.8	19.6	17.8
**Smoking status/dose (%)**									
*Never*	35.6	40.9	34.9	36.1	33.9	33.6	33.3	32.3	33.2
*Former- ≤ 20 cigarettes/day*	26.7	25.6	26.7	27.4	27.7	27.0	26.9	26.3	25.4
*Former- > 20 cigarettes/day*	23.2	15.4	23.1	22.9	25.1	27.0	27.4	28.9	28.8
*Current- ≤ 20 cigarettes/day*	3.2	5.2	3.2	2.9	2.9	2.6	2.2	2.5	2.5
*Current- > 20 cigarettes/day*	2.9	4.4	3.2	2.3	2.4	2.3	2.5	2.6	2.5
*Missing*	8.4	8.6	9.0	8.4	8.0	7.6	7.7	7.5	7.7
**Self-reported overall health (%)**									
*Excellent*	13.0	16.4	11.9	14.0	13.2	12.1	11.8	11.6	8.3
*Very good*	36.3	37.3	35.8	38.3	37.4	36.0	34.9	33.9	30.8
*Good*	35.1	31.7	36.3	34.2	34.7	35.5	36.3	37.2	39.1
*Fair*	11.9	10.8	12.4	10.4	11.3	12.4	13.2	13.0	16.5
*Poor*	1.9	2.2	1.9	1.4	1.7	2.0	2.0	2.3	3.4
*Missing*	1.8	1.7	1.8	1.7	1.8	2.0	1.8	2.0	2.0
**Age at retirement (yrs.; %)**									
*Not retired*	18.6	15.5	16.5	18.7	19.9	20.7	21.7	23.5	25.0
*< 50*	2.2	2.4	2.1	2.1	2.2	2.2	2.2	2.5	2.8
*50 to 55*	5.1	4.9	5.0	4.9	5.1	5.2	5.0	5.5	5.8
*55 to 60*	18.7	17.7	18.8	18.5	18.7	19.7	19.5	19.6	19.0
*60 to 65*	32.3	33.0	33.6	32.6	31.9	31.1	30.4	30.1	29.3
*65 to 70*	15.6	17.7	16.3	15.8	15.2	14.0	14.2	12.9	12.3
*≥ 70*	4.7	6.1	4.8	4.6	4.3	4.5	4.3	3.4	3.1
*Missing*	2.8	2.7	2.9	2.7	2.7	2.7	2.7	2.6	2.8
**Marital status (%)**									
*Married or living as married*	70.7	67.9	75.6	71.1	69.8	69.6	68.5	67.4	62.3
*Widowed*	9.8	11.8	8.5	9.7	9.8	9.4	9.7	9.6	11.0
*Divorced*	12.9	12.9	10.0	12.5	14.0	14.4	15.1	16.3	19.1
*Separated*	1.0	1.0	1.0	1.0	0.9	1.1	1.0	1.1	1.3
*Never married*	5.0	5.8	4.2	5.2	5.0	5.2	5.3	5.1	5.8
*Missing*	0.5	0.6	0.6	0.5	0.6	0.4	0.5	0.5	0.5
**Age at menopause (yrs.; %)**									
*< 40*	6.4	5.9	4.4	6.0	6.8	7.4	7.7	9.6	11.9
*40 to 45*	5.7	6.3	3.9	5.5	6.2	6.3	6.8	6.4	8.5
*45 to 50*	9.0	10.7	6.2	9.2	9.3	9.7	10.3	10.8	12.4
*50 to 55*	12.1	14.8	7.8	12.8	12.9	13.6	13.4	14.3	16.0
*≥ 55*	2.6	3.2	1.7	2.7	3.0	2.8	2.9	3.4	3.4
*Still menstruating*	1.8	1.5	0.8	1.8	2.2	2.4	2.5	3.2	3.8
*Male*	62.1	57.2	74.9	61.7	59.4	57.5	56.0	51.8	43.5
*Missing*	0.4	0.4	0.3	0.3	0.3	0.3	0.3	0.5	0.5

*PI* Pacific Islander, *AI* American Indian, *AN* Alaskan Native, *BMI* body mass index, *HEI* healthy eating index, *WL* weight loss, *hrs*. hours, *yrs.* years; *FUQ*, follow-up questionnaire, *SD* standard deviation

### Frequency of weight loss and all-cause mortality

**Fig. 1:**
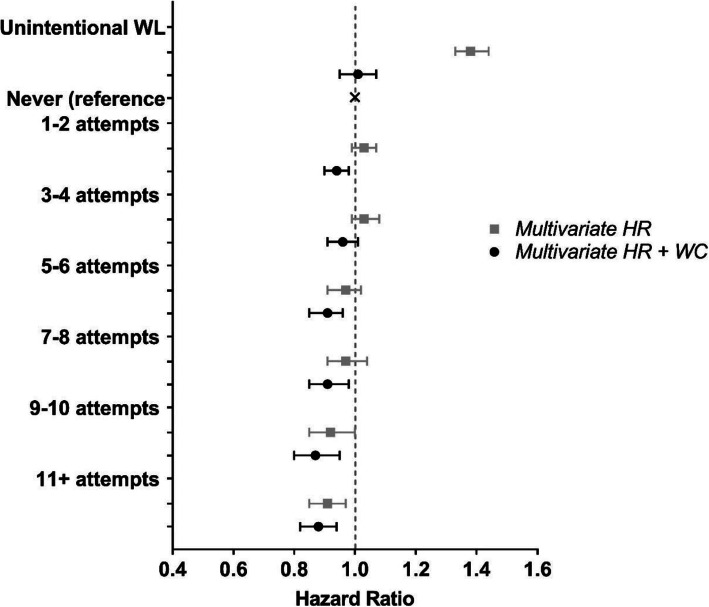
Risk of all-cause mortality associated with frequency of weight loss attempts of at least 5 lbs in the previous 20 years. Multivariate hazard ratios (HRs) are adjusted for age, sex, race/ethnicity, education level, health eating index total score, physical activity, sedentary time, smoking, chronic diseases, self-report overall health, marital status, age at retirement, age at menopause, and starting BMI. Multivariate HRs shown in black are additionally adjusted for weight change (WC) over time

In multivariate models increasing frequency of weight loss of five pounds or more was associated with lower all-cause mortality (*p* trend< 0.0001; Fig. 1; Additional file 1: Table S1). As compared to participants who never attempted weight loss, the highest frequency of weight loss attempts (11+) was associated with a 9% (95% CI, 3–15%) lower risk of all-cause mortality. When the model was further adjusted for weight change over the 20-year period, the associations remained (*p* trend = 0.010) and were stronger across all categories of successful weight loss attempts (e.g., HR = 0.88, 95% CI = 0.82–0. 94 for 11+ attempts).

Those who reported no intentional weight loss attempts but lost weight over the 20-year period (i.e., unintentional weight loss) had higher mortality risk (HR = 1.38, 95% CI = 1.33–1.44) compared to participants who never attempted weight loss but maintained or gained weight. This association was no longer associated after adjustment for weight change categories (HR = 1.01, 95% CI = 0.95–1.07).

**Fig. 2:**
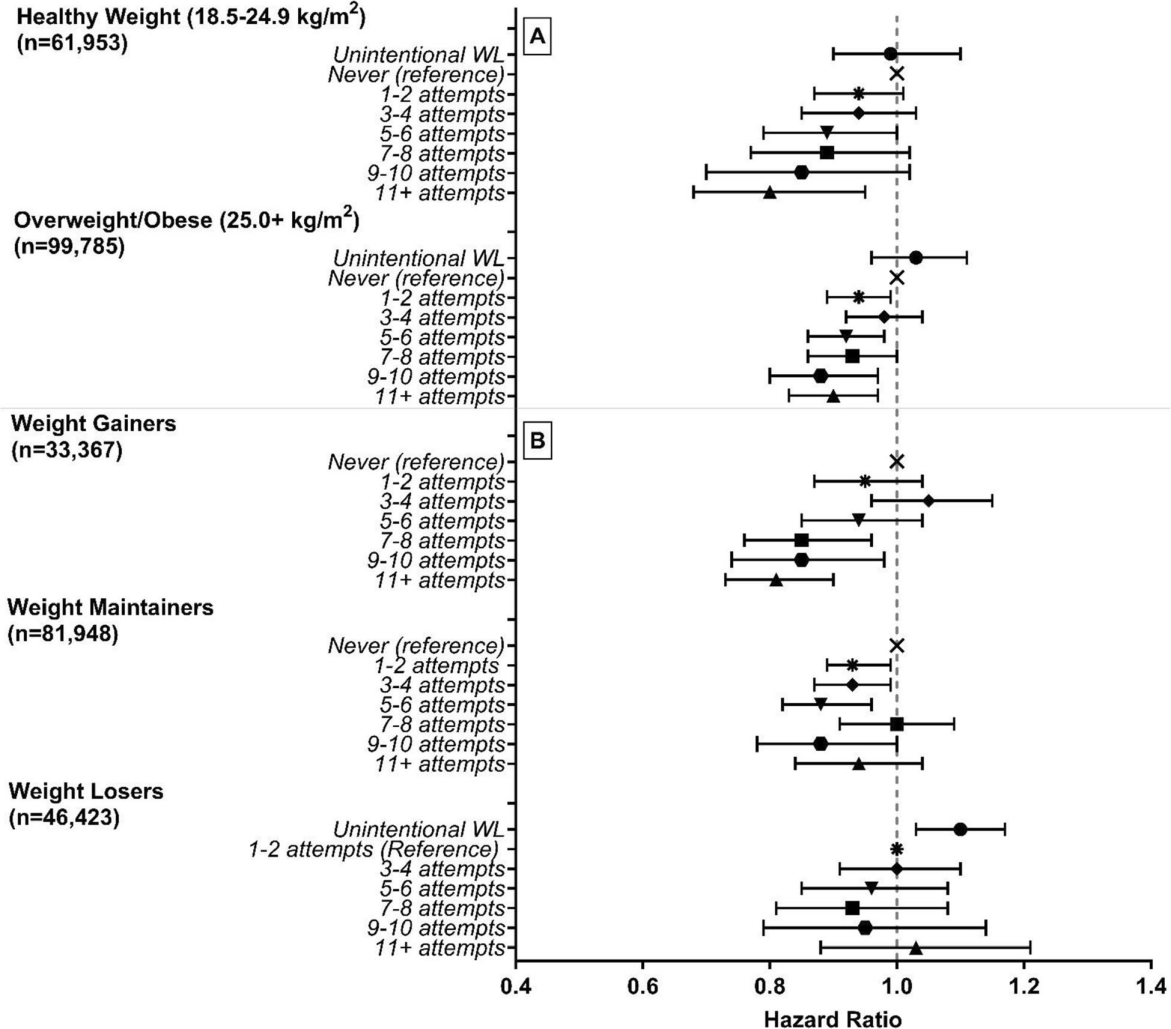
Stratified analysis of proportional hazard ratios for all-cause mortality associated with frequency of weight loss (WL) attempts of at least 5 lbs over the previous 20 years, by (A) historical BMI and (B) life course weight loss. Hazard ratios and 95% confidence intervals (CI) are adjusted for age, sex, race/ethnicity, education level, healthy eating index total score, physical activity, sedentary time, smoking, chronic diseases, self-reported overall health, marital status, age at retirement, age at menopause, starting BMI, and weight change. Weight losers: reference group = 1–2 attempts

The inverse association between weight loss attempts and total mortality was similar when stratified by age (Additional file 1: Table S2), for men and women (Additional file 1: Table S1), and participants who were overweight/obese or had healthy BMI at the beginning of the weight loss period (Fig. 2 (A); Additional file 1: Table S2). Even among those who gained weight over the time period, an inverse association was observed with 19% (95% CI, 10–27%) lower risk of mortality for those with 11+ attempts (Fig. 2 (B) and Additional file 1: Table S2). There were no associations observed for weight maintainers (HR = 0.94, 95% CI 0.84–1.04) or weight losers (HR = 1.03, 95% CI 0.88–1.21) with 11+ weight loss attempts. Across smoking status strata, an inverse association was observed between weight loss attempts and total mortality among never and former smokers, but not current smokers (Additional file 1: Table S2). Results were similar when we used multiple imputation to evaluate that possible impact of excluding participants with missing values on intentional weight loss (Additional file 1: Table S5).

**Fig. 3:**
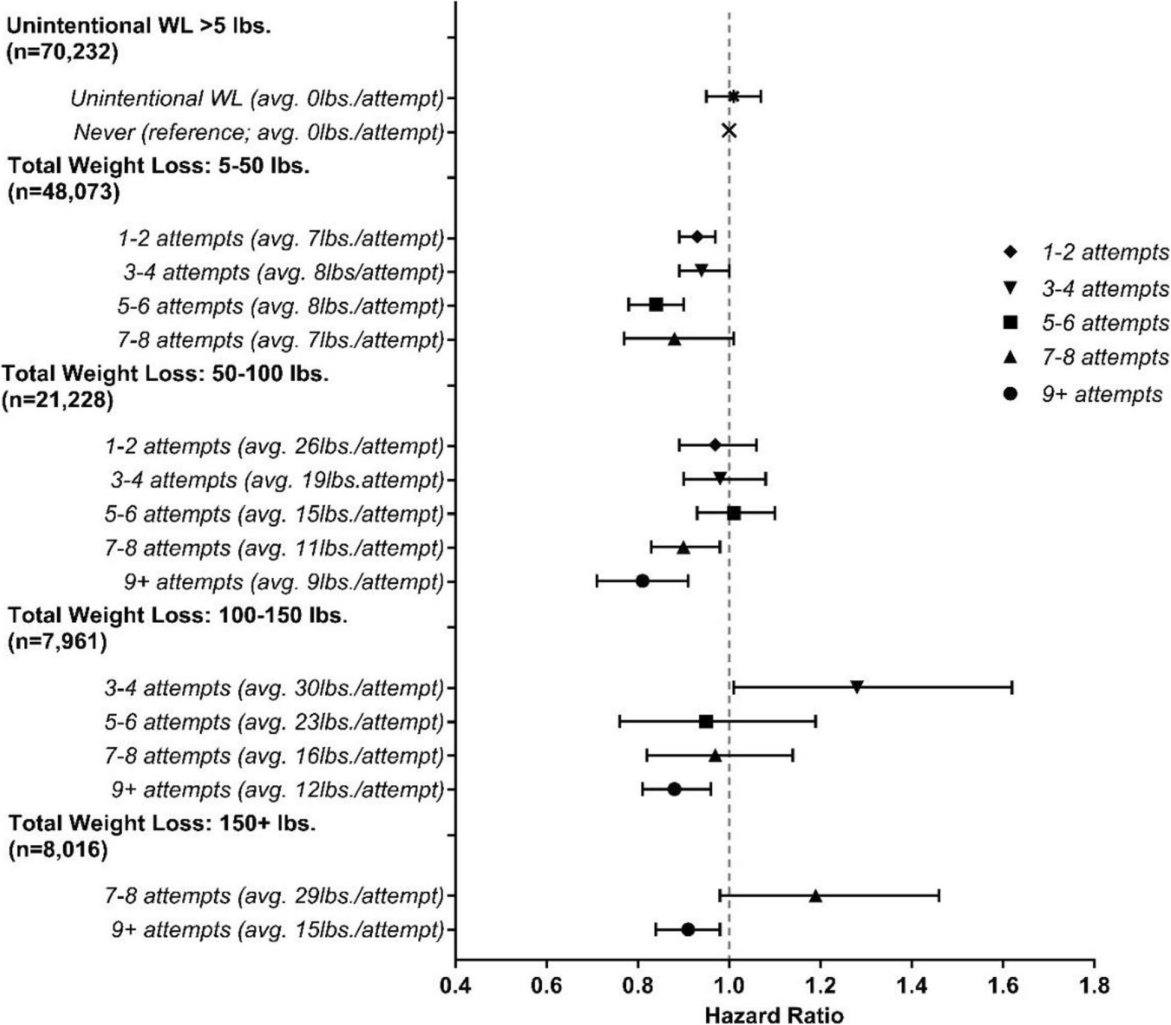
Proportional hazard ratios for all-cause mortality based on the joint effects of the frequency of weight loss attempts and the total weight loss based on the sum of those attempts the previous 20 years (*n* = 161,738). Hazard ratios and 95% confidence intervals (CI) adjusted for age, sex, race/ethnicity, education level, healthy eating index total score, physical activity, sedentary time, smoking, chronic diseases, self-report overall health, marital status, age at retirement, age at menopause, starting BMI, and weight change

To explore whether the observed associations were driven by total volume of weight lost, we jointly classified categories of total weight lost over the 20 years and frequency of those attempts and estimated the average amount of weight loss per attempt. (Fig. 3 and Additional file 1: Table S3). Within most categories of total weight loss, there was a trend toward reduced mortality with increased frequency of attempts. However, there was some evidence that the association between frequency of weight loss and mortality was modified by total weight loss volume. For example, reduced risk was observed in those with 5–6 weight loss attempts who lost 5–50 lbs (HR = 0.84; 95% CI = 0.78–0.90), but no association in those who lost 100–150 lbs with the same number of attempts (HR = 0.95; 95% CI = 0.76–1.19). For large amounts of total weight loss (e.g., 100–150 lbs), more frequent attempts (9+) with more moderate losses per attempt (12 lbs/attempt) were associated with lower risk (HR = 0.91, 95% CI 0.84–0.98), while fewer attempts with larger losses per attempt (30 lbs/attempt) were not (HR = 1.19, 95% CI 0.98–1.46).

### Frequency of weight loss and cause-specific mortality

**Fig. 4:**
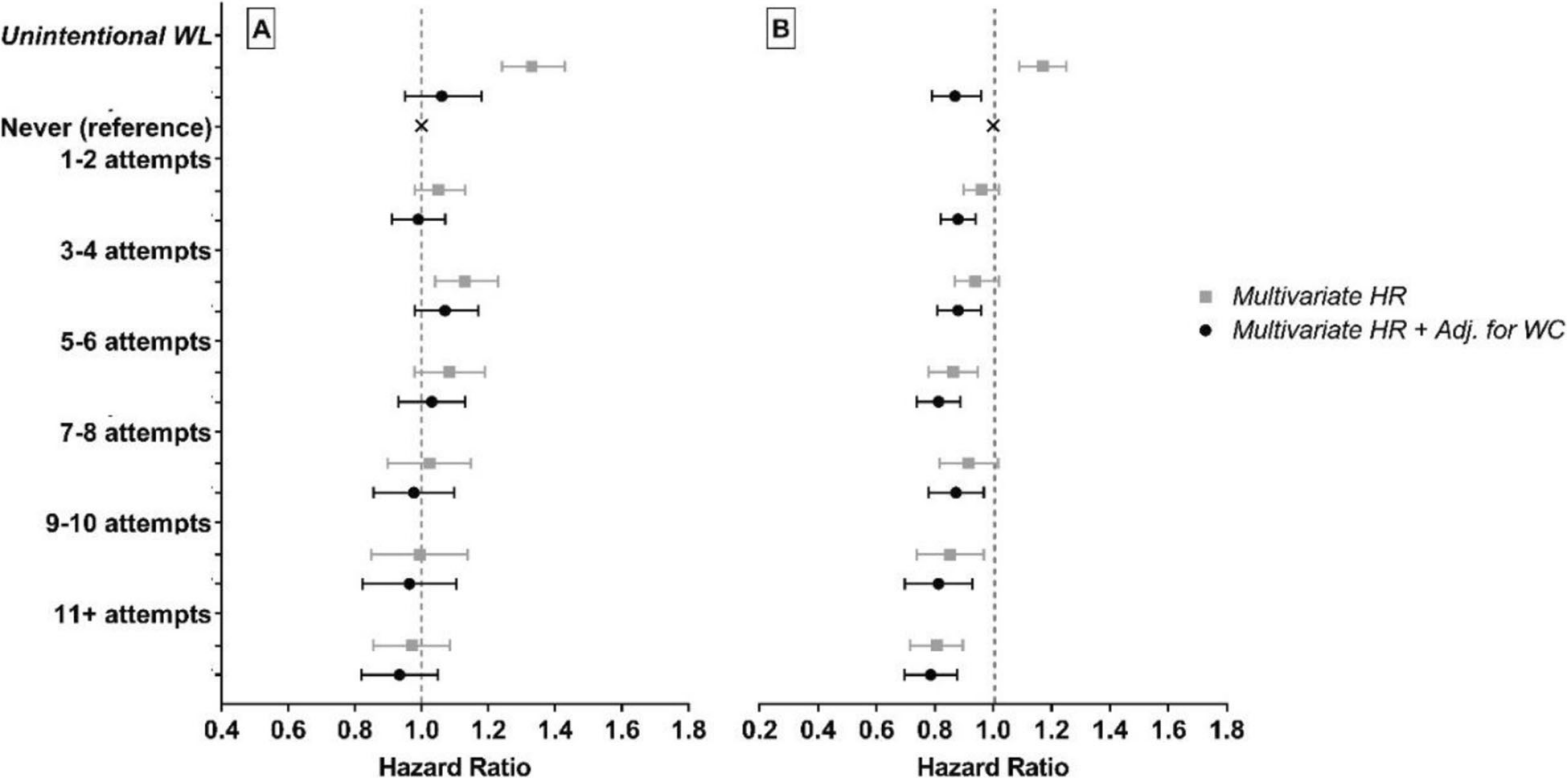
Cardiovascular (A) and cancer (B) specific mortality HRs for frequency of weight loss attempts in the previous 20 years. Multivariate sub-distribution hazard ratios and 95% confidence interval (CI) adjusted for age, sex, race/ethnicity, education level, healthy eating index total score, physical activity, sedentary time, smoking, chronic diseases, self-report overall health, marital status, age at retirement, age at menopause, starting BMI, and weight change (WC)

The dose-response pattern of association with increasing frequency of weight loss attempts was apparent for both cancer mortality (7850 deaths) and cardiovascular mortality (6430 deaths, Fig. 4 and Table Additional file 1: S4). For cancer mortality, there was an inverse association with increasing frequency of weight loss attempts with the highest frequency of weight loss attempts (11+) having a 22% (95% CI, 12–30%) lower risk. For cardiovascular mortality, there was no significant association observed for any frequency group. However, a significant linear trend for both cancer (*p* = 0.004) and CVD mortality (*p* = 0.021; Additional file 1: Table S4) was observed. After additionally controlling for weight change, the linear trends approached significance (both *p* < 0.065; Additional file 1: Table S4).

## Discussion

In this large prospective US cohort, we observed that increased frequency of weight loss attempts of five pounds or more in middle-aged adults was associated with lower future mortality risk. As compared with individuals who never intentionally lost at least five pounds, individuals who had 11+ attempts over 20 years had a 12% lower risk of death. Notably, this inverse association was observed even among those who gained weight over the 20-year period, suggesting some benefit to frequent attempts at weight loss even if weight is eventually gained over time. Inverse associations were also evident in men and women and participants who were initially healthy weight or overweight/obese. Associations were also noted for deaths caused by cardiovascular disease and cancer.

Our findings are consistent with a recent animal study by Smith and colleagues [31], who randomized obese mice to ad libitum feeding to sustain obesity, calorie restriction to achieve a “normal” or intermediate body weight, or weight cycling (repeated episodes of calorie restriction and ad libitum refeeding). As compared to the sustained obesity group, mice who had periodic weight losses showed 43% lower mortality risk. Furthermore, recent studies suggest that periodic fasting cycles play a beneficial role by promoting the activation of degradation and turnover pathways that promote repair and removal of damaged macromolecules in humans [32–35]. Combined with our results, this evidence suggests that middle-aged adults may benefit from repeated weight loss attempts in terms of longevity, even if overall individuals gain weight over time.

Previous studies assessing weight cycling over time and mortality risk have yielded mixed results [16–20, 36, 37]. Studies evaluating weight change based on measured or self-reported weights at discrete time intervals (e.g., biennial visits) have largely observed an increased risk of mortality with fluctuations in weight; however, these studies did not differentiate between intentional and unintentional weight loss and did not evaluate weight fluctuations, including weight loss attempts, between visits or questionnaires. The increased mortality observed in these studies may be more reflective of individuals with episodes of significant unintentional weight loss due to illness and/or those engaging in more extreme dieting. Although the numbers were small in our study, we did not see a mortality benefit for those with large amounts of weight lost (e.g., 100+ lbs) over few attempts, and those with unintentional weight loss were at increased risk of mortality. In addition, variability in covariates adjusted for in previous studies of weight change may also explain differences between these studies and ours. For example, not all studies adjusted for baseline BMI, and others did not adjust for education or other potential confounders. The importance of adjusting for BMI was highlighted by the results by Stevens et al. [37], in which weight cycling appeared harmful in models not adjusted for BMI, but not in models accounting for BMI. We adjusted for a large number of important confounding factors, including BMI, physical activity, and sedentary time, in our analysis, and this may have helped clarify an independent association not seen in previous studies.

In contrast to studies of weight change based on fixed time points, studies evaluating weight cycling based on self-reported intentional weight loss attempts have not observed an increased risk of mortality. Consistent with our study, Field et al. [36] reported a reduced risk of mortality for weight cyclers based on weight loss attempts over 20 years. Similarly, examining only those who purposefully lost and regained 10+ pounds, Stevens et al. [37] found lower mortality for those lost and gained weight 1–4 or 5–9 times, although no association with those who lost and regained weight 20 or more times over their lifetime. Unlike the study by Stevens et al. [37], we did not have data on weight regained after each attempt and so could not examine the number of times that individuals regained the weight lost. While nearly all individuals regain weight previously lost, some do not and those that do may do so at different rates [38]. By adjusting for weight change over the time, we were able to account for the cumulative effects of weight loss and gain in our analysis, even if not the individual effects.

A unique aspect of our analysis is the joint effects models of total volume of weight lost and frequency of attempts. Infrequent attempts with large volumes of weight lost provided no mortality benefit while more frequent attempts with moderate amounts of weight loss per attempt was associated with lower mortality risk. This suggests that repeated moderate amount of weight loss may provide more long-term benefit than losing a large amount of weight all at once. This is consistent with the recommendation that individuals should lose weight gradually (1–2 lbs/week) [9], and the benefit observed for frequent weight loss attempts may be because these individuals engage in healthy behaviors (e.g., exercise, healthy diet, etc.) over a cumulative longer time period than those who made no attempts. The increased risk observed with large losses of weight with infrequent attempts may be explained by possible unhealthy behaviors (e.g., meal skipping, diet pills, laxatives, diuretics, or purging) [39] that individuals may engage in to lose high volumes of weight in a single weight loss attempt. Thus, this study presents a novel analysis addressing the joint effects of total volume of weight lost and frequency of attempts not previously studied. Also, our study differed from previous studies [16–20] that did not differentiate between intentional and unintentional weight loss, investigations [16–20] that failed to adjust for BMI at baseline, and studies [16–20, 36] that did not adjust for other potential confounding variables.

The association between frequency of weight loss attempts and mortality was strongest among individuals who gained weight over time with limited or no benefit for those that lost or maintained their weight. We hypothesize that overall loss or maintenance of weight is the primary driver of longevity. Among those who achieve weight loss or maintenance, the frequency of the number of attempts may be less important because they have been successful in overall reducing or maintaining their weight and thus have gained the benefit in longevity. The frequency of weight loss attempts may be more important for those who ultimately gain weight, as they are at higher risk and do not have the benefit of achieving long-term weight loss or maintenance. The frequency of attempts may be reflective of increased attempts at engaging in healthy behaviors, leading to reduced mortality seen in those ultimately gaining weight.

One potential limitation of our study is that associations could reflect confounding by unmeasured or poorly measured confounders, including other unmeasured behavior changes. Although we cannot rule out residual confounding, the availability of detailed data enabled us to comprehensively adjust for many possible confounders, including BMI and sedentary time. Since past frequency of weight loss attempts was self-reported, some measurement error is inevitable due to inaccurate recall, but this misclassification is likely non-differential and presumably only biased our results toward the null. The subjects were prospectively followed for mortality after completing the questionnaire, so it is unlikely there were any systematic differences in recall between those who later died and those who did not. Second, information on previous weight loss attempts was not available for all participants. Although it is possible that participants missing weight loss information are different than the rest of the cohort, we found that those who were excluded due to missing data were broadly similar in socio-demographics and behavioral characteristics to those who were included, and no differences in mortality were observed based on death records. The results were similar after imputation to account for missing data, suggesting that our findings are valid. Third, we lack data on how and when weight loss was attempted over the 20-year period, thus, we were unable to examine the influence on mortality by weight loss methods (e.g., diet, physical activity, pharmacotherapy, bariatric surgery), timing of weight loss methods, duration of each attempt, or the weight loss attempts over shorter periods of time (e.g., < 20 years). The follow-up time was relatively short for mortality in our study (e.g., 7 years on average), but we had a large number of deaths (*N* = 21,194) giving us substantial power to detect an association. Future studies may benefit from longer follow-up. Finally, the generalizability of our results may be limited because our cohort was primarily composed of non-Hispanic Caucasian middle-aged adults that were highly educated. Therefore, future research is encouraged to investigate these associations in more diverse study populations.

Our prospective study with its large sample is the first to evaluate the long-term effects of both the frequency of intentional weight loss attempts over time and the joint effects of frequency with total weight loss. The protective association observed with increasing frequency of weight loss attempts suggests benefit even for those who have difficulty maintaining weight loss. There are plausible mechanisms by which intentional weight loss may provide longevity benefits. In addition to the increased time living with lower levels of fat mass, healthy weight loss attempts are typically accompanied by changes in other healthy behaviors, specifically better eating behaviors, reduced alcohol consumption, and increased physical activity [10]. We did not observe more healthy behaviors in those with frequent weight loss attempts based on self-reported data, but those changes may have only occurred during the period of a weight loss attempt and therefore may have not been captured by the questionnaire. Regardless if the healthy behaviors were maintained, individuals with more frequent intentional weight loss attempts may have had more total time exposed to these healthy behaviors and less years living with overweight/obesity over a lifetime compared to those never successfully attempting weight loss. In combination, weight loss attempts and these other health behaviors may be important for longevity.

## Conclusion

In this large prospective cohort study, we discovered that more frequent intentional weight loss attempts over a 20-year period in mid-life was associated with a reduced risk of death, even among those who ultimately gained weight. The benefits were more evident among those who lost moderate amounts of weight frequently as opposed to those who underwent a few very large weight losses. If replicated, this finding is of high clinical importance due to the increased prevalence of obesity and the difficulty in maintaining weight loss. Although repeated bouts of weight loss followed by weight regain may not be ideal, they are a common occurrence. Our results suggest that frequent intentional weight loss attempts are not harmful and may provide long-term benefit.

## Supplementary information

**Additional File 1: Fig. S1.** Assessment of frequency and volume of intentional weight loss. **Table S1.** All-Cause Mortality HRs for Frequency of Weight Loss Attempts; overall and by sex. **Table S2.** All-Cause Mortality HRs for Frequency of Weight Loss Attempts; by historical BMI, weight change, age, and smoking status. **Table S3.** Mortality HRs for Joint Effects of Total Weight loss and frequency of Weight Loss Attempts. **Table S4.** Sub-distribution cause-specific Mortality HRs for Frequency of Weight Loss Attempts. **Table S5.** All-Cause Mortality HRs for Frequency of Weight Loss Attempts with imputed missing data. **Table S6.** Comparison of Weight Change Definitions for Stratified All-Cause Mortality HRs for Frequency of Weight Loss Attempts.

## Data Availability

The datasets used and/or analyzed during the current study are available from the National Institutes of Health’s National Cancer Institute.
