# Supplementary material for: Increased frequency of intentional weight loss associated with reduced mortality: a prospective cohort analysis
Source: BMC Med. 2020 Sep 17;18:248. doi: 10.1186/s12916-020-01716-5 (PMC7495833; doi:10.1186/s12916-020-01716-5)
Supplement: Supplementary file 1 — Additional File 1: Fig. S1. Assessment of frequency and volume of intentional weight loss. Table S1. All-Cause Mortality HRs for Frequency of Weight Loss Attempts; overall and by sex. Table S2. All-Cause Mortality HRs for Frequency of Weight Loss Attempts; by historical BMI, weight change, age, and smoking status. Table S3. Mortality HRs for Joint Effects of Total Weight loss and frequency of Weight Loss Attempts. Table S4. Sub-distribution cause-specific Mortality HRs for Frequency of Weight Loss Attempts. Table S5. All-Cause Mortality HRs for Frequency of Weight Loss Attempts with imputed missing data. Table S6. Comparison of Weight Change Definitions for Stratified All-Cause Mortality HRs for Frequency of Weight Loss Attempts. [file 12916_2020_1716_MOESM1_ESM.docx]

**ADDITIONAL FILE 1:** SUPPLEMENTAL TABLES AND FIGURES


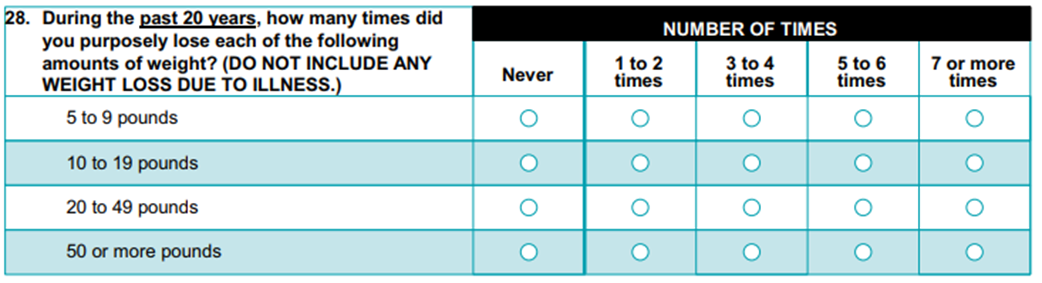


**Figure S1.** Assessment of frequency and volume of intentional weight loss.

| **Table S1. All-Cause Mortality HRs for Frequency of Weight Loss Attempts in the Previous 20 years (AARP Cohort n=161,738)** | | | | | | | | | |
| --- | --- | --- | --- | --- | --- | --- | --- | --- | --- |
|  | **Unintentional**  **+ WL** | **Never (reference)** | **1-2**  **attempts** | **3-4**  **attempts** | **5-6**  **attempts** | **7-8**  **attempts** | **9-10**  **attempts** | **11+**  **attempts** | **p-trend** |
| **All** |  |  |  |  |  |  |  |  |  |
| No. | 26178 | 44054 | 33114 | 19465 | 13537 | 9159 | 6054 | 10177 | - |
| Deaths | 4429 | 6027 | 4014 | 2368 | 1558 | 1046 | 644 | 1108 | - |
| Model 1 HR (95%CI) | 1.38 (1.33-1.44) | 1.00 | 1.03 (0.99-1.07) | 1.03 (0.99-1.08) | 0.97 (0.91-1.02) | 0.97 (0.91-1.04) | 0.92 (0.85-1.00) | 0.91 (0.85-0.97) | <.0001 |
| Model 2 HR (95%CI) | 1.01 (0.95-1.07) | 1.00 | 0.94 (0.90-0.98) | 0.96 (0.91-1.01) | 0.91 (0.85-0.96) | 0.91 (0.85-0.98) | 0.87 (0.80-0.95) | 0.88 (0.82-0.94) | 0.010 |
|  |  |  |  |  |  |  |  |  |  |
| **Males** |  |  |  |  |  |  |  |  |  |
| No. | 14965 | 32979 | 20438 | 11552 | 7785 | 5128 | 3138 | 4431 | - |
| Deaths | 2965 | 4720 | 2778 | 1603 | 1016 | 670 | 387 | 594 | - |
| Model 1 HR (95%CI) | 1.38 (1.32-1.45) | 1.00 | 1.02 (0.97-1.07) | 1.04 (0.98-1.10) | 0.97 (0.91-1.04) | 0.97 (0.91-1.04) | 0.93 (0.84-1.04) | 0.96 (0.88-1.04) | 0.024 |
| Model 2 HR (95%CI) | 0.99 (0.93-1.07) | 1.00 | 0.93 (0.89-0.98) | 0.97 (0.91-1.03) | 0.91 (0.85-0.98) | 0.91 (0.85-0.98) | 0.88 (0.79-0.98) | 0.92 (0.84-1.00) | 0.195 |
|  |  |  |  |  |  |  |  |  |  |
| **Females** |  |  |  |  |  |  |  |  |  |
| No. | 11213 | 11075 | 12676 | 7913 | 5752 | 4031 | 2916 | 5746 | - |
| Deaths | 1464 | 1307 | 1236 | 765 | 542 | 376 | 257 | 514 | - |
| Model 1 HR (95%CI) | 1.37 (1.27-1.48) | 1.00 | 1.04 (0.97-1.13) | 1.02 (0.93-1.12) | 0.96 (0.86-1.06) | 0.97 (0.86-1.09) | 0.90 (0.79-1.03) | 0.85 (0.76-0.94) | 0.0002 |
| Model 2 HR (95%CI) | 1.04 (0.94-1.16) | 1.00 | 0.95 (0.87-1.03) | 0.95 (0.86-1.04) | 0.89 (0.81-0.99) | 0.92 (0.81-1.03) | 0.86 (0.75-0.98) | 0.82 (0.74-0.91) | 0.0094 |
| **Model 1:** adjusted for age, sex, Race/Ethnicity, education level, HEI total score, physical activity, sedentary time, smoking, chronic diseases, self-report overall health, marital status, age at retirement, Age at Menopause, starting BMI | | | | | | | | | |
|  |  |  |  |  |  |  |  |  |  |
| **Model 2:** model 1 adjusted for Weight change | | | | | | | | | |
| HR=hazard ratio, and CI=confidence interval, WL=weight loss | | | | | | | | | |

| **Table S2. Stratified All-Cause Mortality HRs for Frequency of Weight Loss Attempts in the Previous 20 years stratified by BMI, weight change groups, age, and smoking status** | | | | | | | | | |
| --- | --- | --- | --- | --- | --- | --- | --- | --- | --- |
|  | **Unintentional + WL** | **Never (reference)** | **1-2 attempts** | **3-4 attempts** | **5-6 attempts** | **7-8 attempts** | **9-10 attempts** | **11+ attempts** | **p-trend** |
| **Healthy Weight (18.5-24.9 kg/m^2^; n=61,953)** |  |  |  |  |  |  |  |  |  |
| No. | 13762 | 18586 | 12729 | 6622 | 4018 | 2649 | 1504 | 2083 |  |
| Deaths | 1963 | 2245 | 1262 | 614 | 359 | 226 | 119 | 152 |  |
| HR (95% CL) | 0.99 (0.90-1.10) | 1.00 | 0.94 (0.87-1.01) | 0.94 (0.85-1.03) | 0.89 (0.79-1.00) | 0.89 (0.77-1.02) | 0.85 (0.70-1.02) | 0.80 (0.68-0.95) | 0.005 |
| **Overweight/Obese (25.0+ kg/m^2^; n=99,785)** |  |  |  |  |  |  |  |  |  |
| No. | 12416 | 25468 | 20385 | 12843 | 9519 | 6510 | 4550 | 8094 |  |
| Deaths | 2466 | 3782 | 2752 | 1754 | 1199 | 820 | 525 | 956 |  |
| HR (95% CL) | 1.03 (0.96-1.11) | 1.00 | 0.94 (0.89-0.99) | 0.98 (0.92-1.04) | 0.92 (0.86-0.98) | 0.93 (0.86-1.00) | 0.88 (0.80-0.97) | 0.90 (0.83-0.97) | 0.002 |
| **Weight Losers (n=46,423)** |  |  |  |  |  |  |  |  |  |
| No. | 4429 | - | 1307 | 608 | 367 | 210 | 124 | 165 |  |
| Deaths | 26178 | - | 9488 | 4415 | 2645 | 1639 | 977 | 1081 |  |
| HR (95% CL) | 1.10 (1.03-1.17) | - | 1.00 | 1.00 (0.91-1.10) | 0.96 (0.85-1.08) | 0.93 (0.81-1.08) | 0.95 (0.79-1.14) | 1.03 (0.88-1.21) | 0.065 |
| **Weight Maintainers (n=81,948)** |  |  |  |  |  |  |  |  |  |
| No. | - | 35075 | 17485 | 10569 | 7098 | 4693 | 2907 | 4121 |  |
| Deaths | - | 4585 | 1881 | 1110 | 701 | 498 | 275 | 415 |  |
| HR (95% CL) | - | 1.00 | 0.93 (0.89-0.99) | 0.93 (0.87-0.99) | 0.88 (0.82-0.96) | 1.00 (0.91-1.09) | 0.88 (0.78-1.00) | 0.94 (0.84-1.04) | 0.375 |
| **Weight Gainers (n=33,367)** |  |  |  |  |  |  |  |  |  |
| No. | - | 8979 | 6141 | 4481 | 3794 | 2827 | 2170 | 4975 |  |
| Deaths | - | 1442 | 826 | 650 | 490 | 338 | 245 | 528 |  |
| HR (95% CL) | - | 1.00 | 0.95 (0.87-1.04) | 1.05 (0.96-1.15) | 0.94 (0.85-1.04) | 0.85 (0.76-0.96) | 0.85 (0.74-0.98) | 0.81 (0.73-0.90) | <0.001 |
| **Age <51 years (n=80,972)** |  |  |  |  |  |  |  |  |  |
| No. | 9832 | 19707 | 16633 | 10517 | 7825 | 5504 | 3952 | 7002 |  |
| Deaths | 1016 | 1671 | 1158 | 783 | 544 | 392 | 295 | 535 |  |
| HR (95% CL) | 1.03 (0.92-1.16) | 1.00 | 0.87 (0.81-0.95) | 0.93 (0.85-1.01) | 0.83 (0.75-0.91) | 0.83 (0.74-0.93) | 0.88 (0.78-1.00) | 0.83 (0.75 0.92) | 0.042 |
| **Age ≥51 years (n=80,766)** |  |  |  |  |  |  |  |  |  |
| No. | 16346 | 24347 | 16481 | 8948 | 5712 | 3655 | 2102 | 3175 |  |
| Deaths | 3413 | 4356 | 2856 | 1585 | 1014 | 654 | 349 | 573 |  |
| HR (95% CL) | 1.01 (0.95-1.09) | 1.00 | 0.97 (0.92-1.02) | 0.98 (0.92-1.02) | 0.95 (0.89-1.02) | 0.97 (0.89-1.05) | 0.86 (0.77-0.96) | 0.90 (0.83-0.99) | <0.001 |
| **Never Smokers (n=57,552)** |  |  |  |  |  |  |  |  |  |
| No. | 10696 | 15362 | 11965 | 6603 | 4543 | 3045 | 1956 | 3382 |  |
| Deaths | 1150 | 1316 | 965 | 548 | 362 | 250 | 153 | 270 |  |
| HR (95% CL) | 1.02 (0.96-1.08) | 1.00 | 0.93 (0.90-0.98) | 0.97 (0.92-1.02) | 0.92 (0.86-0.97) | 0.92 (0.86-0.99) | 0.88 (0.81-0.96) | 0.89 (0.83-0.95) | 0.747 |
| **Former Smokers (n=80,727)** |  |  |  |  |  |  |  |  |  |
| No. | 10729 | 21911 | 16673 | 10275 | 7312 | 4979 | 3342 | 5506 |  |
| Deaths | 2145 | 3485 | 2257 | 1340 | 934 | 619 | 389 | 634 |  |
| HR (95% CL) | 1.00 (0.92-1.08) | 1.00 | 0.91 (0.86-0.96) | 0.90 (0.84-0.96) | 0.87 (0.81-0.94) | 0.87 (0.79-0.95) | 0.83 (0.75-0.93) | 0.83 (0.76-0.90) | 0.001 |
| **Current Smokers (n=9,937)** |  |  |  |  |  |  |  |  |  |
| No. | 2509 | 2800 | 1706 | 1022 | 658 | 434 | 304 | 504 |  |
| Deaths | 686 | 605 | 354 | 235 | 122 | 71 | 55 | 96 |  |
| HR (95% CL) | 0.97 (0.81-1.16) | 1.00 | 0.98 (0.85-1.14) | 1.19 (1.01-1.40) | 0.91 (0.74-1.11) | 0.85 (0.66-1.09) | 0.97 (0.73-1.28) | 1.03 (0.82-1.29) | 0.233 |
| **Note:** Hazard ratios (HRs) are adjusted for age, sex, race/ethnicity, education level, healthy eating index total score, physical activity, sedentary time, smoking, chronic diseases, self-report overall health, marital status, age at retirement, age at menopause, starting BMI and weight change, as appropriate. HR=Hazard Ratio, CL=confidence limits. | | | | | | | | | |
|  |  |  |  |  |  |  |  |  |  |
|  |  |  |  |  |  |  |  |  |  |

| **Table S3. Mortality HRs for Joint Effects of Total Weight loss and frequency of Weight Loss Attempts of in the Previous 20 years** | | | | | | | | | |  |  |  |  |
| --- | --- | --- | --- | --- | --- | --- | --- | --- | --- | --- | --- | --- | --- |
|  | **Unintentional WL** |  | **Never (reference)** |  | **1-2 attempts** |  | **3-4 attempts** |  | **5-6 attempts** |  | **7-8 attempts** |  | **9+ attempts** |
| **Never Purposefully losing at least 5lbs.** |  |  |  |  |  |  |  |  |  |  |  |  |  |
| *No.* | 26178 |  | 44054 |  | - |  | - |  | - |  | - |  | - |
| *Deaths* | 4429 |  | 6027 |  | - |  | - |  | - |  | - |  | - |
| *HR (95%CI)* | 1.01 (0.95-1.07) |  | 1.00 |  | - |  | - |  | - |  | - |  | - |
| *Average weight loss/attempt (lbs.)* | 0 |  | 0 |  | - |  | - |  | - |  | - |  | - |
| *Total Weight loss over 20 years (lbs.)* | 0 |  | 0 |  | - |  | - |  | - |  | - |  | - |
|  |  |  |  |  |  |  |  |  |  |  |  |  |  |
| **Total Weight Loss: 5-50lbs.** | **Unintentional WL** |  | **Never** |  | **1-2 attempts** |  | **3-4 attempts** |  | **5-6 attempts** |  | **7-8 attempts** |  | **9+ attempts** |
| *No.* | - |  | - |  | 25390 |  | 13392 |  | 7388 |  | 1903 |  | - |
| *Deaths* | - |  | - |  | 3384 |  | 1775 |  | 854 |  | 215 |  | - |
| *HR (95%CI)* | - |  | - |  | 0.93 (0.89-0.97) |  | 0.94 (0.89-1.99) |  | 0.84 (0.78-0.90) |  | 0.88 (0.77-1.01) |  | - |
| *Average weight loss/attempt (lbs.)* | - |  | - |  | 6.9 ± 2.5 |  | 8.4 ± 2.4 |  | 8.4 ± 1.4 |  | 7.0 ± 0.1 |  | - |
| *Total Weight loss over 20 years (lbs.)* | - |  | - |  | 13.8 ± 4.9 |  | 28.6 ± 4.1 |  | 44.2 ± 4.0 |  | 48.9 ± 0.4 |  | - |
|  |  |  |  |  |  |  |  |  |  |  |  |  |  |
| **Total Weight Loss: 50-100lbs.** | **Unintentional WL** |  | **Never** |  | **1-2 attempts** |  | **3-4 attempts** |  | **5-6 attempts** |  | **7-8 attempts** |  | **9+ attempts** |
| *No.* | - |  | - |  | 4340 |  | 3891 |  | 4573 |  | 5593 |  | 2831 |
| *Deaths* | - |  | - |  | 630 |  | 512 |  | 603 |  | 623 |  | 267 |
| *HR (95%CI)* | - |  | - |  | 0.97 (0.89-1.06) |  | 0.98 (0.90-1.08) |  | 1.00 (0.93-1.10) |  | 0.90 (0.83-0.98) |  | 0.81 (0.71-0.91) |
| *Average weight loss/attempt* | - |  | - |  | 26.0 ± 0.0 |  | 18.6 ± 4.9 |  | 15.1 ± 1.8 |  | 11.0 ± 2.3 |  | 8.6 ± 1.0 |
| *Total Weight loss over 20 years* | - |  | - |  | 52.0 ± 0.0 |  | 60.8 ± 9.0 |  | 78.0 ± 9.6 |  | 77.0 ± 16.1 |  | 77.5 ± 9.6 |
|  |  |  |  |  |  |  |  |  |  |  |  |  |  |
| **Total Weight Loss: 100-150lbs.** | **Unintentional WL** |  | **Never** |  | **1-2 attempts** |  | **3-4 attempts** |  | **5-6 attempts** |  | **7-8 attempts** |  | **9+ attempts** |
| *No.* | - |  | - |  | - |  | 369 |  | 601 |  | 1029 |  | 5962 |
| *Deaths* | - |  | - |  | - |  | 69 |  | 78 |  | 143 |  | 651 |
| *HR (95%CI)* | - |  | - |  | - |  | 1.28 (1.01-1.62) |  | 0.95 (0.76-1.19) |  | 0.97 (0.82-1.14) |  | 0.88 (0.81-0.96) |
| *Average weight loss/attempt* | - |  | - |  | - |  | 30.3 ± 0.0 |  | 23.3 ± 3.3 |  | 15.5 ± 1.8 |  | 11.8 ± 1.7 |
| *Total Weight loss over 20 years* | - |  | - |  | - |  | 121.0 ± 0.0 |  | 116.3 ± 16.7 |  | 112.1 ± 10.1 |  | 122.0 ± 14.8 |
|  |  |  |  |  |  |  |  |  |  |  |  |  |  |
| **Total Weight Loss: 150+lbs.** | **Unintentional WL** |  | **Never** |  | **1-2 attempts** |  | **3-4 attempts** |  | **5-6 attempts** |  | **7-8 attempts** |  | **9+ attempts** |
| *No.* | - |  | - |  | - |  | - |  | - |  | 578 |  | 7438 |
| *Deaths* | - |  | - |  | - |  | - |  | - |  | 100 |  | 834 |
| *HR (95%CI)* | - |  | - |  | - |  | - |  | - |  | 1.19 (0.98-1.46) |  | 0.91 (0.84-0.98) |
| *Average weight loss/attempt* | - |  | - |  | - |  | - |  | - |  | 29.0 ± 8.0 |  | 14.8 ± 3.6 |
| *Total Weight loss over 20 years* | - |  | - |  | - |  | - |  | - |  | 187.9 ± 40.6 |  | 214.8 ± 69.2 |
| **Note:** Hazard ratios (HRs) are adjusted for age, sex, Race/Ethnicity, education level, HEI total score, physical activity, sedentary time, smoking, chronic diseases, self-report overall health, marital status, age at retirement, Age at Menopause, starting BMI, and weight change. CI=confidence Interval. | | | | | | | | | | | | | |
|  |  |  |  |  |  |  |  |  |  |  |  |  |  |

| **Table S4. Sub-distribution cause-specific Mortality HRs for Frequency of Weight Loss Attempts in the Previous 20 years (Total Sample n=161,738)** | | | | | | | | | |
| --- | --- | --- | --- | --- | --- | --- | --- | --- | --- |
|  | **Unintentional WL** | **Never (reference)** | **1-2 attempts** | **3-4 attempts** | **5-6 attempts** | **7-8 attempts** | **9-10 attempts** | **11+ attempts** | **p-trend** |
| **Cancer Mortality** |  |  |  |  |  |  |  |  |  |
| No. | 26178 | 44054 | 33114 | 19465 | 13537 | 9159 | 6054 | 10177 | - |
| Deaths | 1534 | 2374 | 1517 | 872 | 553 | 391 | 234 | 375 | - |
| Model 1 HR (95%CI) | 1.17 (1.09-1.25) | 1.00 | 0.96 (0.90-1.02) | 0.94 (0.87-1.02) | 0.86 (0.78-0.95) | 0.91 (0.82-1.02) | 0.85 (0.74-0.97) | 0.80 (0.72-0.90) | 0.004 |
| Model 2 HR (95%CI) | 0.87 (0.79-0.96) | 1.00 | 0.88 (0.82-0.94) | 0.88 (0.81-0.96) | 0.81 (0.74-0.89) | 0.87 (0.78-0.97) | 0.81 (0.70-0.93) | 0.78 (0.70-0.88) | 0.057 |
|  |  |  |  |  |  |  |  |  |  |
| **CVD Mortality** |  |  |  |  |  |  |  |  |  |
| No. | 14965 | 32979 | 20438 | 11552 | 7785 | 5128 | 3138 | 4431 | - |
| Deaths | 1247 | 1802 | 1225 | 769 | 516 | 324 | 204 | 343 | - |
| Model 1 HR (95%CI) | 1.33 (1.24-1.43) | 1.00 | 1.05 (0.98-1.13) | 1.13 (1.04-1.23) | 1.08 (0.98-1.19) | 1.02 (0.90-1.15) | 0.99 (0.85-1.14) | 0.96 (0.86-1.09) | 0.021 |
| Model 2 HR (95%CI) | 1.06 (0.95-1.18) | 1.00 | 0.99 (0.91-1.07) | 1.07 (0.98-1.17) | 1.03 (0.93-1.13) | 0.97 (0.86-1.10) | 0.95 (0.82-1.10) | 0.93 (0.82-1.05) | 0.065 |
| **Model 1:** adjusted for age, sex, Race/Ethnicity, education level, HEI total score, physical activity, sedentary time, smoking, chronic diseases, self-report overall health, marital status, age at retirement, Age at Menopause, starting BMI | | | | | | | | | |
|  |  |  |  |  |  |  |  |  |  |
| **Model 2:** model 1 adjusted for Weight change | | | | | | | | | |
| CVD=cardiovascular disease, WL=weight loss, HR=hazard ratio, and CI=confidence interval | | | | | | | | | |

| **Table S5. All-Cause Mortality HRs for Frequency of Weight Loss Attempts in the Previous 20 Years including AARP participants with Missing Data (n=268,756)** | | | | | | | | |  |
| --- | --- | --- | --- | --- | --- | --- | --- | --- | --- |
|  | **Unintentional + WL** | **Never (reference)** | **1-2 attempts** | **3-4 attempts** | **5-6 attempts** | **7-8 attempts** | **9-10 attempts** | **11+ attempts** | **p-trend** |
| **Analysis Cohort** | 1.02 (0.96-1.07) | 1.00 | 0.96 (0.93-1.00) | 0.97 (0.93-1.02) | 0.95 (0.89-1.01) | 0.95 (0.88-1.02) | 0.93 (0.86-0.99) | 0.93 (0.87-0.98) | <.0001 |
| **Sex** |  |  |  |  |  |  |  |  |  |
| *Males* | 1.01 (0.95-1.08) | 1.00 | 0.96 (0.92-1.01) | 0.98 (0.93-1.03) | 0.95 (0.88-1.03) | 0.95 (0.86-1.05) | 0.94 (0.85-1.04) | 0.95 (0.87-1.02) | 0.009 |
| *Females* | 1.02 (0.92-1.14) | 1.00 | 0.97 (0.90-1.05) | 0.97 (0.89-1.06) | 0.94 (0.85-1.04) | 0.95 (0.84-1.07) | 0.91 (0.81-1.02) | 0.89 (0.82-0.98) | <0.001 |
| **BMI Category** |  |  |  |  |  |  |  |  |  |
| *Healthy Weight (18.5-24.9 kg/m^2^)* | 1.01 (0.92-1.11) | 1.00 | 0.96 (0.90-1.04) | 0.97 (0.90-1.05) | 0.93 (0.84-1.04) | 0.94 (0.83-1.07) | 0.90 (0.73-1.09) | 0.91 (0.78-1.06) | 0.003 |
| *Overweight/Obese (25.0+ kg/m^2^)* | 1.03 (0.97-1.10) | 1.00 | 0.97 (0.93-1.02) | 0.99 (0.94-1.04) | 0.96 (0.90-1.03) | 0.97 (0.89-1.05) | 0.95 (0.88-1.03) | 0.95 (0.89-1.01) | 0.077 |
| **Weight Change group** |  |  |  |  |  |  |  |  |  |
| *Weight Losers* | 1.06 (1.00-1.13) | - | 1.00 | 1.00 (0.91-1.09) | 0.97 (0.87-1.09) | 0.95 (0.82-1.10) | 0.96 (0.80-1.14) | 1.05 (0.90-1.22) | 0.007 |
| *Weight Maintainers* | - | 1.00 | 0.96 (0.91-1.02) | 0.96 (0.90-1.01) | 0.94 (0.86-1.02) | 0.99 (0.90-1.09) | 0.94 (0.84-1.05) | 0.95 (0.87-1.05) | 0.251 |
| *Weight Gainers* | - | 1.00 | 0.98 (0.90-1.06) | 1.03 (0.93-1.14) | 0.97 (0.88-1.07) | 0.93 (0.84-1.03) | 0.91 (0.80-1.03) | 0.88 (0.80-0.97) | 0.002 |
| **Age Group** |  |  |  |  |  |  |  |  |  |
| *<51 years* | 1.03 (0.93-1.15) | 1.00 | 0.93 (0.86-1.00) | 0.96 (0.88-1.05) | 0.90 (0.80-1.01) | 0.91 (0.81-1.02) | 0.94 (0.81-1.08) | 0.89 (0.81-0.99) | <0.001 |
| *≥51 years* | 1.02 (0.96-1.08) | 1.00 | 0.98 (0.94-1.03) | 0.98 (0.93-1.04) | 0.98 (0.91-1.04) | 0.98 (0.90-1.06) | 0.91 (0.83-1.00) | 0.94 (0.87-1.02) | 0.006 |
| **Smoking Status** |  |  |  |  |  |  |  |  |  |
| *Never Smokers* | 1.04 (0.94-1.16) | 1.00 | 0.98 (0.91-1.06) | 1.02 (0.92-1.12) | 1.00 (0.88-1.14) | 1.01 (0.87-1.17) | 0.99 (0.83-1.18) | 1.01 (0.90-1.14) | 0.363 |
| *Former Smokers* | 1.01 (0.94-1.08) | 1.00 | 0.95 (0.90-0.99) | 0.94 (0.89-0.99) | 0.93 (0.86-1.01) | 0.92 (0.85-1.00) | 0.90 (0.82-0.99) | 0.89 (0.82-0.96) | <0.001 |
| *Current* | 0.98 (0.83-1.16) | 1.00 | 0.98 (0.86-1.12) | 1.12 (0.96-1.29) | 0.90 (0.76-1.08) | 0.88 (0.70-1.12) | 1.02 (0.80-1.29) | 0.99 (0.80-1.23) | 0.488 |
| *Note:* Results from multiple imputation analyses. Model was adjusted for age, sex, Race/Ethnicity, education level, HEI total score, physical activity, sedentary time, smoking, chronic diseases, self-report overall health, marital status, age at retirement, Age at Menopause, starting BMI, and weight change | | | | | | | | |  |

| **Table S6. Comparison of Weight Change Definitions for Stratified All-Cause Mortality HRs for Frequency of Weight Loss Attempts in the Previous 20 years stratified** | | | | | | | | | |
| --- | --- | --- | --- | --- | --- | --- | --- | --- | --- |
|  | **Unintentional + WL** | **Never (reference)** | **1-2 attempts** | **3-4 attempts** | **5-6 attempts** | **7-8 attempts** | **9-10 attempts** | **11+ attempts** | **p-trend** |
| **DEFINED BY SLOPE WEIGHT CHANGE** |  |  |  |  |  |  |  |  |  |
| **Weight Losers (n=46,423)** |  |  |  |  |  |  |  |  |  |
| No. | 4429 | - | 1307 | 608 | 367 | 210 | 124 | 165 | - |
| Deaths | 26178 | - | 9488 | 4415 | 2645 | 1639 | 977 | 1081 | - |
| HR (95% CL) | 1.10 (1.03-1.17) | - | 1.00 | 1.00 (0.91-1.10) | 0.96 (0.85-1.08) | 0.93 (0.81-1.08) | 0.95 (0.79-1.14) | 1.03 (0.88-1.21) | 0.065 |
| **Weight Maintainers (n=81,948)** |  |  |  |  |  |  |  |  |  |
| No. | - | 35075 | 17485 | 10569 | 7098 | 4693 | 2907 | 4121 | - |
| Deaths | - | 4585 | 1881 | 1110 | 701 | 498 | 275 | 415 | - |
| HR (95% CL) | - | 1.00 | 0.93 (0.89-0.99) | 0.93 (0.87-0.99) | 0.88 (0.82-0.96) | 1.00 (0.91-1.09) | 0.88 (0.78-1.00) | 0.94 (0.84-1.04) | 0.375 |
| **Weight Gainers (n=33,367)** |  |  |  |  |  |  |  |  |  |
| No. | - | 8979 | 6141 | 4481 | 3794 | 2827 | 2170 | 4975 | - |
| Deaths | - | 1442 | 826 | 650 | 490 | 338 | 245 | 528 | - |
| HR (95% CL) | - | 1.00 | 0.95 (0.87-1.04) | 1.05 (0.96-1.15) | 0.94 (0.85-1.04) | 0.85 (0.76-0.96) | 0.85 (0.74-0.98) | 0.81 (0.73-0.90) | <0.001 |
|  |  |  |  |  |  |  |  |  |  |
| **DEFINED AS ±3%** |  |  |  |  |  |  |  |  |  |
| **Weight Losers (n=63,914)** |  |  |  |  |  |  |  |  |  |
| No. | 28408 | - | 12890 | 7553 | 5243 | 3461 | 2340 | 4019 | - |
| Deaths | 4821 | - | 1786 | 1001 | 651 | 438 | 278 | 464 |  |
| HR (95% CL) | 1.10 (1.04 1.16) | - | 1.00 | 0.97 (0.90 1.05) | 0.91 (0.83 1.00) | 0.92 (0.82 1.02) | 0.89 (0.79 1.01) | 0.89 (0.79 1.01) | 0.008 |
| **Weight Maintainers (n=29,483)** |  |  |  |  |  |  |  |  |  |
| No. | - | 15402 | 5852 | 3205 | 1938 | 1302 | 815 | 969 | - |
| Deaths | - | 2091 | 676 | 377 | 222 | 153 | 89 | 136 | - |
| HR (95% CL) | - | 1.00 | 0.93 (0.85 1.02) | 0.90 (0.80 1.00) | 0.90 (0.78 1.04) | 0.94 (0.79 1.11) | 0.84 (0.67 1.04) | 0.94 (0.78 1.13) | 0.823 |
| **Weight Gainers (n=68,341)** |  |  |  |  |  |  |  |  |  |
| No. | - | 26422 | 14372 | 8707 | 6356 | 4396 | 2899 | 5189 | - |
| Deaths | - | 3544 | 1552 | 990 | 685 | 455 | 277 | 508 | - |
| HR (95% CL) | - | 1.00 | 0.91 (0.86 0.97) | 0.98 (0.92 1.06) | 0.89 (0.82 0.97) | 0.88 (0.80 0.98) | 0.82 (0.73 0.93) | 0.82 (0.74 0.91) | 0.002 |
| **Note:** Hazard ratios (HRs) are adjusted for age, sex, race/ethnicity, education level, healthy eating index total score, physical activity, sedentary time, smoking, chronic diseases, self-report overall health, marital status, age at retirement, age at menopause, starting BMI and weight change, as appropriate. HR=Hazard Ratio, CL=confidence limits. | | | | | | | | | |
|  |  |  |  |  |  |  |  |  |  |
|  |  |  |  |  |  |  |  |  |  |
